# Bridging Surgical Outcomes and Patient Experience: A Questionnaire-Based Evaluation of Buccal Mucosal Graft Morbidity Following Substitution Urethroplasty

**DOI:** 10.7759/cureus.94647

**Published:** 2025-10-15

**Authors:** Zakaria W Shkoukani, Abdul Rauf, Mohamed Abdulmajed, Ahmad Omar, Michael St Floyd

**Affiliations:** 1 Department of Reconstructive Urology, Mersey and West Lancashire Teaching Hospitals NHS Trust, Prescot, GBR

**Keywords:** buccal mucosal grafting, patient-reported outcome measures, patient’s satisfaction, post-operative morbidity, urethroplasty

## Abstract

Introduction

Urethroplasty remains the gold standard for managing complex urethral stricture disease (USD). Buccal mucosa grafts (BMG) are the preferred graft material due to low morbidity and ease of harvest. However, specific donor site morbidity remains underexplored. This study prospectively evaluates oral morbidity following BMG harvest through use of a dedicated questionnaire.

Methods

All patients undergoing urethral reconstruction with BMG were prospectively enrolled. Demographic data, stricture characteristics, graft dimensions and operative details were recorded. At postoperative day 10, a six-item questionnaire was utilised to assess oral pain scores, resumption of normal diet, paraesthesia, trismus, and overall patient satisfaction. Data was analysed using descriptive and inferential statistics.

Results

Sixty-four BMGs were harvested, including three bilateral cases. Procedures included bulbar urethroplasties (n = 28, 43.7%), staged graft urethroplasties (n = 23, 35.9%), augmented perineal urethrostomies (n = 8, 12.5%), Asopa technique urethroplasties (n = 4, 6.3%), and one meatoplasty (1.6%). Median stricture length was 2 cm; median graft dimensions were 4 x 1.5 cm (L x W). Most patients (n = 58, 90.6%) reported pain scores ≤ 2 out of 10, and 54 patients (84.3%) resumed normal diet by postoperative day two. Oral morbidity was transient, with 13 (20.3%) experiencing trismus, 11 (17.2%) reporting paraesthesia, and two (3.1%) having xerostomia, all resolving by day 10. All patients expressed willingness to undergo BMG harvesting again if required. No statistically significant association was found between graft characteristics and morbidity.

Conclusion

BMG harvesting in our unit is associated with low morbidity and high patient satisfaction. A dedicated questionnaire effectively captures patient-reported outcomes, supporting ongoing quality improvement.

## Introduction

Urethroplasty is recognised as the gold standard treatment for both primary and recurrent urethral stricture disease, particularly for long-segment strictures, owing to its superior durability, high long-term success rates, and lower recurrence compared with endoscopic interventions [1]. Various substitution techniques have been developed over time, including ventral onlay, dorsal inlay, and other modified approaches [2]. The choice of technique is primarily determined by the stricture’s location, length, and underlying aetiology, in addition to the surgeon’s expertise and familiarity with specific reconstructive approaches [3].

A range of graft materials have been employed in substitution urethroplasty, including bladder mucosa, penile and scrotal skin grafts, lingual mucosa, tunica vaginalis flaps, and acellular dermal matrix grafts [4-8]. However, buccal mucosa has emerged as the optimal graft material due to its favourable structural and functional characteristics, including ease of harvest, low donor-site morbidity, resilience to desiccation, and excellent tissue integration [9].

While most existing literature has predominantly focused on functional outcomes such as postoperative voiding and erectile function, few studies have systematically assessed graft-site morbidity using validated patient-reported outcome measures [10,11].

The primary objective of this study was to prospectively evaluate oral morbidity following buccal mucosal graft harvesting using a structured qualitative questionnaire. The secondary objective was to identify the most common postoperative symptoms and assess their impact on oral function and patient quality of life in the early postoperative period at a high-volume reconstructive urology centre in the United Kingdom [10].

## Materials and methods

Inclusion criteria

All male patients who underwent substitution urethroplasty with buccal mucosal grafting for anterior urethral stricture disease at our institution between September 2017 and September 2025 were prospectively enrolled in this study. No exclusion criteria were applied in the selection of participants.

Pre-operative assessment and surgical planning

Patients diagnosed with urethral stricture disease were evaluated in a dedicated specialist stricture clinic. A comprehensive medical history was obtained, with specific attention to established risk factors such as prior urethral instrumentation, perineal or pelvic trauma, sexually transmitted infections, and a history of paediatric urethral surgery. Exclusion criteria for buccal mucosal grafting included active oral infections or inflammatory mucosal conditions (such as lichen planus, candidiasis, gingivitis, periodontitis, or ulceration) and any history of oral malignancy treated with surgical resection or radiotherapy, due to the associated risks of impaired graft viability, compromised mucosal integrity, and delayed wound healing. Urethroplasty was not performed in patients with distal urethral strictures who were active smokers, due to the risk of poor wound healing and graft failure associated with tobacco use [12]. For patients presenting with proximal (bulbar) urethral strictures, detailed preoperative counselling and smoking cessation interventions were implemented to reduce the risk of graft-related complications.

Patients were also screened for the feasibility of nasal intubation; contraindications included a history of nasal or midfacial trauma (notably Le Fort II or III fractures), severe septal deviation, or obstructive nasal pathology. Physical examination encompassed an evaluation of the oral cavity, buccal mucosa, maximal jaw opening, as well as a focused urogenital and perineal assessment. All patients were weighed at their initial visit and underwent both flexible cystoscopy and urethrography by the operating surgeon to permit detailed stricture characterisation.

Each case was reviewed in a regional reconstruction multidisciplinary team (MDT) meeting to determine the most appropriate surgical intervention. Informed consent was obtained pre-operatively, with specific discussion of graft harvest site complications, including potential for infection, bleeding, fibrosis, iatrogenic injury to Stensen’s duct, and oral paraesthesia. Separate written informed consent was obtained for intraoperative medical photography.

Harvesting technique

The choice between single-stage and two-stage urethroplasty was guided by the stricture’s location, length, and aetiology. In general, single-stage urethroplasty was preferred for short bulbar urethral strictures, whereas two-stage urethroplasty was reserved for complex or extensive distal strictures associated with significant fibrosis or severe spongiofibrosis. All graft procedures were performed by one reconstructive urologist. Nasotracheal intubation was utilised in all cases.

**Figure 1:**
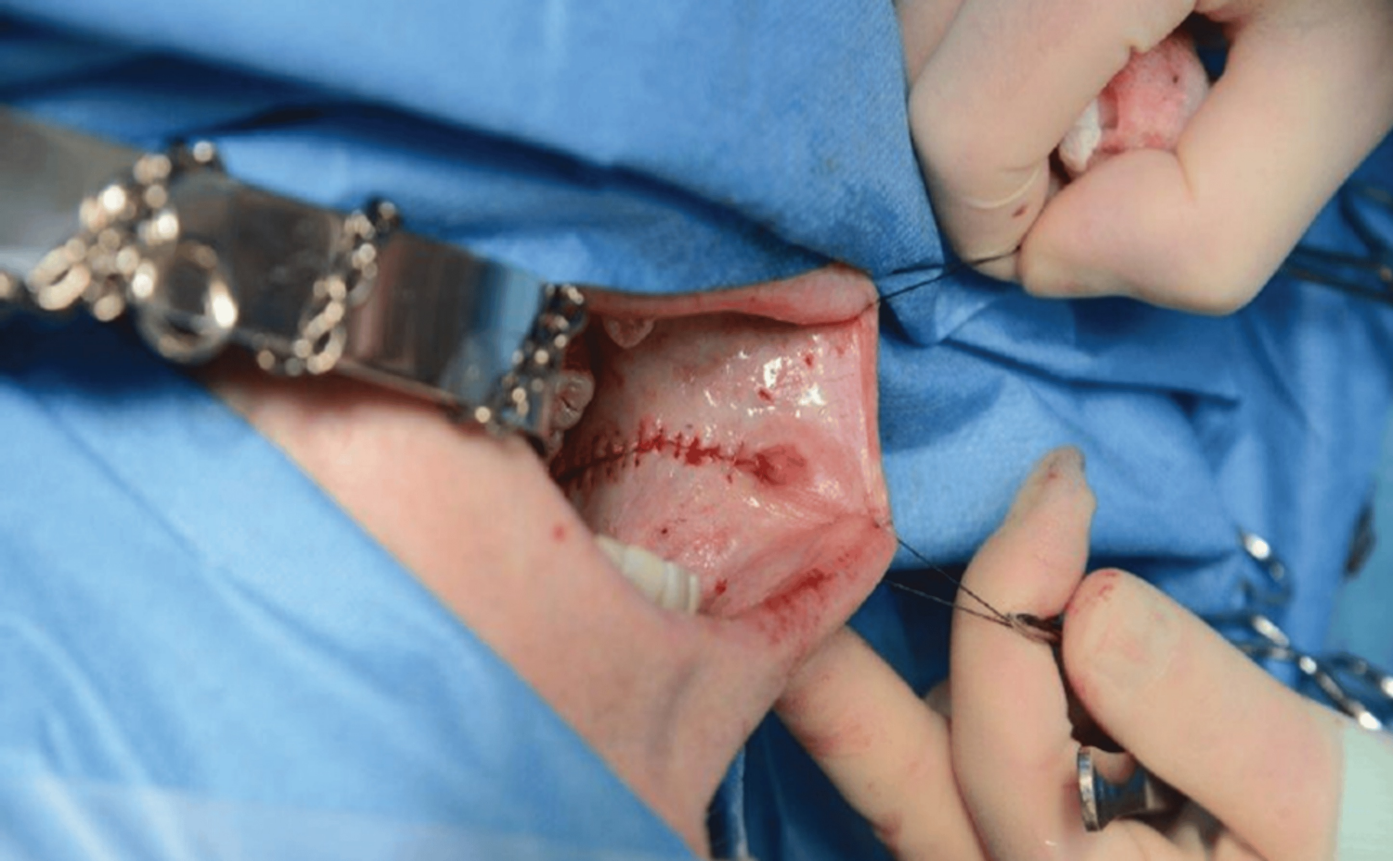
Primary closure of buccal donor site following graft harvesting.

**Figure 2:**
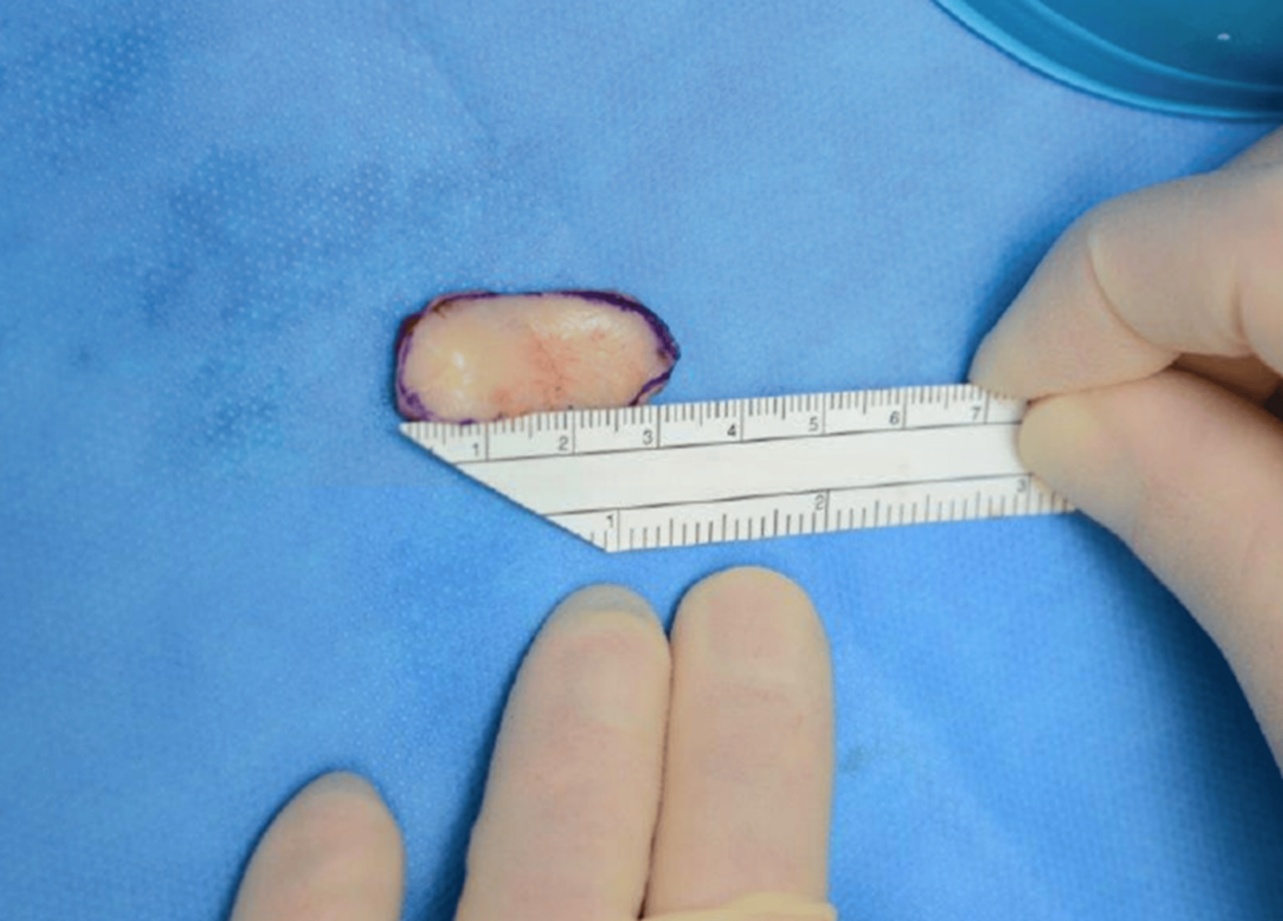
Buccal mucosal graft with measured dimensions.

The oral cavity was prepared for graft harvesting with McKesson’s mouth retractors. The donor site was carefully delineated and secured with two stay sutures, ensuring a minimum distance of 1 cm away from Stensen’s duct (identified by means of a lacrimal probe and intraoperative photography). Infiltration with diluted local anaesthetic (1:200,000) facilitated tissue dissection with minimal trauma to the underlying buccinator. Following stricture length assessment, a bespoke buccal mucosal graft was then meticulously dissected and harvested from the oral cavity. Haemostasis was achieved using diathermy cauterisation only when necessary, and the donor site was primarily closed in continuous fashion with absorbable 4/0 Vicryl suture material. Intraoperative photographs were obtained to confirm wound closure and preservation of Stensen’s duct anatomy (Figure 1). Separate photographs of the graft were taken to document graft size and dimensions (Figure 2).

Follow-up

All patients followed a standardised postoperative care protocol. Patients were prescribed oral Benzydamine mouthwash for five days postoperatively and allowed unrestricted intake of clear fluids on the evening of surgery, followed by the reintroduction of a normal diet on the first postoperative day. All distal urethral grafts were given 24 hours of intravenous antibiotics followed by four further days of oral treatment. Bulbar and perineal cases were given 48 hours of intravenous antibiotics, followed by three days of oral treatment. Patients who underwent a staged urethroplasty for distal urethral strictures were discharged on postoperative day one with an indwelling catheter in place. Those undergoing repair for bulbar urethral strictures or perineal urethrostomy were discharged with a catheter on postoperative day two.

On day 10 all graft patients were assessed for signs of submucosal haematoma, oedema, impacted speech or jaw opening, or evidence of infection. Patients were then requested to complete a dedicated qualitative questionnaire to subjectively evaluate donor site morbidity.

Questionnaire

A structured six-item qualitative questionnaire was used to systematically assess oral morbidity following buccal mucosal graft harvesting [10]. As outlined in Appendix 1, the questionnaire studied the following key parameters: number of days taken for the resumption of a normal diet and full mouth opening, numerical oral pain scores on postoperative day 10, changes in saliva production, the presence of oral paraesthesia, overall patient satisfaction with the procedure and the perceived duration of oral wound healing.

Data collection and analysis

All data were prospectively collected and subsequently subjected to retrospective analysis. Demographic information, stricture aetiology, and stricture length as determined by retrograde urethrography were extracted from the local electronic patient record system (Careflow EPR, System C Healthcare Ltd, Stratford-upon-Avon, UK). Operative notes were reviewed using the local electronic theatre system (OPERA software) to obtain procedural details, including the type of urethroplasty performed, as well as graft-specific characteristics such as anatomical harvest site and graft dimensions. Postoperative complications, where present, were documented. Information was abstracted using a standardized data collection form and entered into Microsoft Excel (Microsoft Corp., Redmond, WA, USA), stored securely on encrypted institutional servers accessible only to the research team. Identifiable information was anonymized prior to analysis to maintain participant confidentiality. Data analysis was performed using IBM SPSS Statistics (Version 30; IBM Corp., Armonk, NY, USA). Descriptive and inferential statistics were used to assess postoperative outcomes.

## Results

A total of 64 patients who met the inclusion criteria were enrolled in the study. The median age of participants was 55 years (range: 23-87 years). The median length of anterior urethral stricture was 2 cm (range: 0.5-9 cm), as determined by retrograde urethrography. The aetiological distribution of urethral stricture disease is summarised in Table 1.

**Table 1:** Aetiologies of urethral stricture disease. TURP: Transurethral resection of prostate, *: Endoscopy for any reason other than TURP (e.g. cystoscopy, bladder tumour resection, ureteroscopy, etc).

Aetiology	n	%
Lichen sclerosus	30	46.8
Post TURP	7	11.0
Previous urethral instrumentation ^*^	3	4.6
History of paediatric urethral surgery	3	4.6
Catheter-related causes	2	3.1
Penile cutaneous horn	1	1.8
Idiopathic	18	28.1

**Figure 3:**
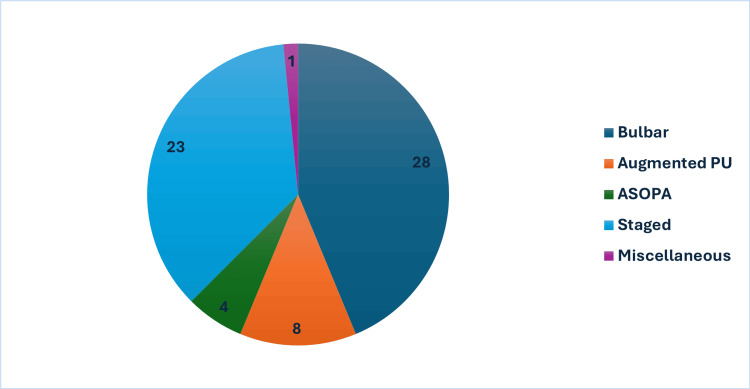
Types of urethroplasty procedures performed. PU: perineal urethrostomy, ASOPA: Asopa technique (dorsal inlay).

The types of urethral graft procedures performed are depicted in Figure 3, with the majority of patients (n=28, 43.7%) undergoing single-stage bulbar substitution urethroplasty. Staged procedures included first-stage, intermediate augmentation, and second-stage urethroplasties; only stages involving buccal mucosal grafting were included in the final count. One miscellaneous case involved a revision meatoplasty incorporating buccal mucosal grafting, performed for severe lichen sclerosus affecting the distal urethra and meatus.

Buccal mucosal grafts were harvested from the right cheek in 49 (76.5%) patients. The left cheek was utilised in eight cases (12.5%); six patients had previously undergone right cheek grafting during a prior first-stage procedure, and in two patients, localisation of Stensen’s duct on the right side proved challenging. Bilateral buccal grafts were required in six patients (9.4%) due to longer grafts being required. In two cases (3.1%), augmentation with an additional lingual graft was performed.

The median graft length across all procedure types was 4 cm (range: 1-10 cm), and the median graft width was 1.5 cm (range: 1-5 cm). All donor sites were closed primarily, except for one case in which the graft dimensions precluded tension-free closure.

Appendix 1 presents the six-item structured questionnaire administered at the postoperative day 10 review to subjectively assess buccal mucosal graft site morbidity [10]. Patient response rates and corresponding percentages are reported in Table 2.

**Table 2:** Dedicated six-item questionnaire with patient responses and percentages.

Question	Response	n	%
Rate the severity of pain inside your mouth using a pain scale ranging from ‘no pain’ (0) to ‘very severe pain’ (10).	≤ 2	58	90.6
	3-5	6	9.4
	≥ 6	0	0
When did you resume your normal conventional diet?	≤ 2 days	54	84.3
	3-5 days	7	11.0
	≥ 6 days	3	4.7
When could you open your mouth as wide as you did before the operation?	≤ 2 days	36	56.2
	3-5 days	15	23.5
	≥ 6 days	13	20.3
Have you felt any numbness inside your mouth?	Yes	11	17.2
	No	53	82.8
Have you experienced a dry mouth since your procedure?	Yes	2	3.1
	No	62	96.9
If needed, would you consider undergoing the same procedure again?	Yes	64	100.0
	No	0	0.0

Notably, among patients who experienced pain persisting beyond postoperative day two (n = 6, 9.4%), the use of benzydamine mouthwash provided adequate analgesic control, and all were pain-free by the day 10 follow-up. One patient demonstrated pre-existing difficulty with mouth opening prior to urethroplasty, which may have influenced their subjective assessment. Of the patients who reported postoperative oral numbness (n = 11, 17.2%), symptoms resolved within 48 hours in all but two cases.

Patient satisfaction was assessed via item 6 of the structured questionnaire. All respondents (n = 64) indicated they would consider buccal mucosal grafting again if clinically required. No donor site complications were identified in our cohort on long-term follow-up. No damage to Stensen’s duct was reported.

Statistical analysis was conducted using a two-tailed, two-sample t-test assuming equal variances, and was performed with Microsoft Excel. No statistically significant associations were found between increasing graft dimensions (length or width, analysed separately) and postoperative pain scores (t-value = 0.031, p-value = 0.487), restoration of full mouth opening (t-value = -0.776, p-value = 0.220), or resumption of a conventional diet (t-value = -0.313, p-value = 0.378). Similar results were observed when comparing outcomes between patients undergoing bilateral versus unilateral graft harvesting.

## Discussion

Buccal mucosa in reconstructive urethral surgery was first documented by Sapezhko in 1894 [13]. Humby in 1941 reported the use of buccal mucosa for the repair of a failed hypospadias in an eight-year-old patient, marking one of the earliest instances of its clinical use in urethral reconstruction [14]. The technique gained widespread recognition in 1993 when El-Kasaby popularised the concept of grafting in urethroplasty [15].

Buccal mucosa is now considered the gold standard graft material for the management of urethral stricture disease. As an autograft it has favourable histological and mechanical properties, including ease of harvesting, resistance to wet environments, and adaptability. Common donor sites for graft harvesting include the inner cheek (buccal mucosa), mandibulo-alveolar region, and lower lip [4,9].

Despite its widespread use, buccal mucosa harvesting has complications. These include postoperative bleeding, submucosal haematoma formation, infection, pain, trismus, and localised oral numbness [16-18].

The incidence and severity of these complications are influenced by several factors: the anatomical site of the graft harvest, graft dimensions, and the method of wound closure - whether primary closure or healing by secondary intention [19].

Studies that advocated for leaving the donor site wound open or avoided the use of diathermy for haemostasis - due to concerns regarding postoperative contracture formation and nerve injury - have reported higher rates of bleeding, particularly within paediatric populations [19-21]. Reported incidences of postoperative bleeding range from 1% to 21% [20,21]. Notably, several of these studies employed the lower lip as the graft harvest site, a factor that may have contributed to the increased complication rates. In a systematic review by Markiewicz et al., grafts harvested from the lower lip were associated with a higher incidence of donor site morbidity compared to those harvested from the buccal mucosa [22]. In our series, grafts were exclusively harvested from the buccal mucosa. Diathermy was used conservatively to achieve haemostasis. A strict oral wound postoperative protocol was adhered to. Using this approach, no cases of submucosal haematoma or secondary haemorrhage were observed in our cohort.

Although specific data regarding the incidence of donor site infections or the necessity for antibiotic prophylaxis remain limited, there is a general consensus in the literature supporting routine postoperative oral hygiene, standardised analgesic protocols, and the administration of prophylactic antibiotics in accordance with local antimicrobial stewardship guidelines [23]. All patients in our study received a 24-48 hour perioperative course of intravenous antibiotics followed by a three- to four-day course of oral antibiotics postoperatively. Additionally, patients were instructed to use benzydamine mouthwash. No cases of donor site infection or dehiscence were identified in our cohort.

Barbagli et al. reported that both large unilateral (defined as >4 cm in length and >2.5 cm in width) and bilateral buccal mucosal graft harvests were associated with significantly higher postoperative pain levels and a delayed return to normal diet compared to smaller unilateral grafts (<4 cm in length and <2.5 cm in width) [18]. Wood et al. demonstrated that leaving the donor site wound to heal by secondary intention, without occlusive dressings, may reduce postoperative discomfort and expedite normal dietary resumption [24]. However, findings from the present study did not demonstrate a statistically significant association between graft size (unilateral versus bilateral, or small versus large) and subjective pain scores. Only six patients (9.4%) reported pain scores exceeding 2 out of 10, and all patients experienced complete symptom resolution by postoperative day 10. Primary closure using a continuous suture technique was performed in all cases except one, where graft dimensions precluded tension-free closure. No cases of contracture were observed. By the second postoperative day, 84.3% of patients had resumed a normal diet (n = 54).

Postoperative oral numbness has been reported in up to 57% of patients undergoing oral mucosal grafting [25]. In our cohort, 11 patients (17.2%) reported transient oral paraesthesia. Symptom resolution occurred within 48 hours in all but two cases (3.1%). The remaining two patients reported complete resolution of paraesthesia by the 10-day postoperative review. These findings are consistent with data from Barbagli et al., who noted that 73% of patients experienced oral numbness limited to the first postoperative week, with full resolution thereafter [18].

In a study by Kulkarni et al., Stensen’s duct injury and subsequent stenosis were reported in two of 256 patients (0.8%) following mucosal graft harvesting [26]. Similarly, Barbagli et al. found that 97.1% of patients experienced no alteration in salivation following the procedure [18]. In our cohort, only two patients (3.1%) reported symptoms of xerostomia, and no cases of injury to Stensen’s duct occurred.

In accordance with findings reported by Bozkurt et al., all patients in our cohort (n = 64) expressed satisfaction with the buccal mucosal graft harvesting procedure and indicated willingness to undergo a similar procedure in the future, should it be required [10].

Although our results align with previously published data and underscore the importance of using a structured, procedure-specific questionnaire to evaluate patient-reported outcomes, several limitations should be acknowledged. First, the sample size was relatively small, which may limit the statistical power and generalisability of the findings. Second, while the structured questionnaire provided valuable insights into patient-reported oral morbidity, it has not yet been formally validated, which may introduce measurement bias. Third, although all patients were followed clinically for more than two years after urethral reconstruction, the dedicated oral morbidity assessment was conducted at postoperative day 10, focusing primarily on short-term donor-site outcomes. This design was intentional to capture acute oral discomfort, pain, and functional limitation during the early recovery phase, rather than long-term sequelae. Finally, as all procedures were performed by a single reconstructive surgeon, the potential for operator-related bias cannot be excluded, which may limit the external reproducibility of the results across different surgical settings. Larger, multicentre prospective studies employing validated, standardised assessment tools and longer-term donor-site follow-up are warranted to confirm these findings and to further characterise the risk profile associated with buccal mucosal graft harvesting. Such data would facilitate the development and clinical adoption of validated instruments for routine assessment of patient-reported outcomes in this context [27-29].

In future work, we aim to incorporate the Boomerang technique, as described in the literature, to optimise graft length on a single side, thereby obviating the need for bilateral buccal mucosal graft harvesting [30].

## Conclusions

In our prospective cohort, buccal mucosa graft harvesting was associated with minimal short-term oral morbidity and high patient satisfaction. The use of a structured questionnaire provided valuable insight into patient-reported outcomes, warranting further validation and longer-term follow-up to confirm these findings. In recent years, increasing attention has been directed toward outcome measures in urethral reconstruction, particularly regarding postoperative erectile function, lower urinary tract symptom improvement, and overall quality of life. With the advent of newer surgical techniques and the growing emphasis on patient-centered outcomes, the evaluation of oral graft donor sites is likely to evolve into a standard component of postoperative assessment in urethral reconstructive surgery.

## Appendices

Appendix 1

Used with permission from Bozkurt et al. [10]

Oral morbidity questionnaire items:

Q1. Rate the severity of pain inside your mouth using a pain scale ranging from ‘no pain’ (0) to ‘very severe pain’ (10). Circle an answer.

0        1       2       3       4       5       6       7       8       9       10

Q2. When did you resume your normal conventional diet? 

- By day 2 post operatively

- Days 3 - 5 post operatively

- Days 6 - 10 post operatively

- I still have difficulties

Q3. When could you open your mouth as wide as you did before the operation?

- By day 2 post operatively

- Days 3 - 5 post operatively

- Days 6 - 10 post operatively

- I still have difficulties

Q4. Have you felt any numbness inside your mouth?

- Yes (if so, how long for? ______________)

- No

Q5. Have you experienced a dry mouth since your procedure?

- Yes (if so, how long for? ______________)

- No

Q6. If needed, would you consider undergoing the same procedure again?

- Yes

- No
